# Late Diagnosis of Unroofed Coronary Sinus Associated with Persistent Left Superior Vena Cava in Patient with Repaired Tetralogy of Fallot: Case Report

**DOI:** 10.3390/life16020342

**Published:** 2026-02-16

**Authors:** Oana Gheorghe-Fronea, Mircea Robu, Sebastian Onciul, Claudia Nica, Cristian Voica, Robert Țigănașu, Gabriel-Petre Gorecki, Horațiu Moldovan

**Affiliations:** 1 Faculty of Medicine, “Carol Davila” University of Medicine and Pharmacy, 050474 Bucharest, Romania; 2 Department of Cardiology, Clinical Emergency Hospital Bucharest, 014461 Bucharest, Romania; 3 Department of Cardiovascular Surgery, Clinical Emergency Hospital Bucharest, 014461 Bucharest, Romania; 4 Department of Anesthesia and Intensive Care, Faculty of Medicine, Titu Maiorescu University, 040051 Bucharest, Romania; 5 Academy of Romanian Scientists, 050711 Bucharest, Romania

**Keywords:** unroofed coronary sinus, persistent left superior vena cava, tetralogy of fallot, congenital heart disease, atrial-level shunt, adult congenital heart disease, multimodality imaging

## Abstract

**Background:** An unroofed coronary sinus (UCS) is a rare congenital cardiac anomaly, accounting for less than 1% of atrial septal defects and frequently associated with a persistent left superior vena cava (PLSVC). Its coexistence with Tetralogy of Fallot (TOF) is exceptionally uncommon and has been reported almost exclusively in isolated case reports. **Case Presentation:** We report the case of a 20-year-old woman with a history of surgically corrected TOF in infancy, who presented with progressive exertional dyspnea. Multimodality imaging, including transthoracic echocardiography and cardiac magnetic resonance imaging, revealed a large atrial-level shunt caused by a type I unroofed coronary sinus associated with a persistent left superior vena cava, leading to significant right-sided chamber dilation and pulmonary hypertension. Notably, this anomaly had not been identified at the time of the initial TOF repair. The patient underwent a successful surgical correction with interatrial compartmentation and tricuspid annuloplasty, with an uneventful postoperative course. **Conclusions:** This case underscores the extreme rarity of the UCS–TOF association and highlights the potential for UCSs with PLSVC to remain clinically silent for years after complex congenital heart surgery. A comprehensive reassessment of the systemic venous and atrial anatomy using advanced multimodality imaging is essential in symptomatic adults with repaired TOF, as late-presenting venous anomalies may have significant hemodynamic and clinical implications.

## 1. Introduction

The coronary sinus (CS) is a venous structure of the heart that embryologically derives from the common cardinal vein [1]. The coronary sinus is the main venous drainage pathway of the heart, collecting the majority of venous blood from the myocardium and returning it to the right atrium, thereby playing a central role in cardiac venous circulation [2]. An unroofed CS is a rare congenital anomaly that represents less than 1% of all congenital disorders and is not an actual atrial septal defect (ASD) but a mere communication between the coronary sinus and the left atrium. Four anatomical types of unroofed coronary sinus have been described: type I, complete unroofing associated with a persistent left superior vena cava (PLSVC); type II, complete unroofing without a persistent left superior vena cava; type III, partial unroofing of the mid portion of the coronary sinus; and type IV, partial unroofing of the terminal portion [3]. Most unroofed coronary sinus defects occur in the complete subtype associated with PLSVC [4].

The association between an unroofed coronary sinus (UCS) and Tetralogy of Fallot (TOF) is exceedingly rare and has been reported only sporadically in the literature [5,6]. To date, there is a single published report describing this association in an adult patient, whereas the available evidence in the pediatric population is limited to a few isolated case reports. These pediatric cases consistently describe UCS in conjunction with TOF as part of a more complex constellation of atrial and systemic venous anomalies rather than as an isolated association, underscoring both the rarity and heterogeneity of this combination.

We present the case of a 20-year-old woman with a history of surgically corrected Tetralogy of Fallot (TOF) at 7 months of age, who was subsequently found to have an unroofed coronary sinus (UCS) associated with a persistent left superior vena cava (PLSVC)—type I. This anomaly had not been identified at the time of the initial TOF repair, illustrating that a UCS may remain occult despite a prior complex congenital cardiac surgery.

## 2. Case Report

We present the case of a 20-year-old woman admitted to our department with exertional dyspnea that developed during the last three months. The patient had a history of double outlet right ventricle and ventricular septal defects (Tetralogy of Fallot) previously treated with a surgical closure with a Teflon patch, infundibular resection, and infundibuloplasty with a pericardial patch at 7 months of age

The cardiovascular examination revealed a regular rate and rhythm, with a prominent grade 3/6 systolic ejection murmur best heard along the left parasternal border, associated with a wide, fixed splitting of the second heart sound. Blood pressure was within normal limits in both upper limbs, with no significant inter-arm difference. Peripheral oxygen saturation in room air was 90%. Jugular venous pressure was normal, and there were no clinical signs of decompensated heart failure, including the absence of peripheral edema. An examination of the respiratory, abdominal, neurological, and musculoskeletal systems was unremarkable, with no additional pathological findings.

Laboratory investigations revealed a mild anemia, with a hemoglobin level of 10 g/dL, in the absence of leukocytosis and with normal inflammatory markers. Liver biochemistry demonstrated mild hepatic cytolysis, with aspartate aminotransferase (AST) 68 U/L and alanine aminotransferase (ALT) 74 U/L. Renal function tests showed mild azotemia, with a serum creatinine level of 1.6 mg/dL and blood urea nitrogen of 58 mg/dL, consistent with a mild impairment of renal function.

**Figure 1:**
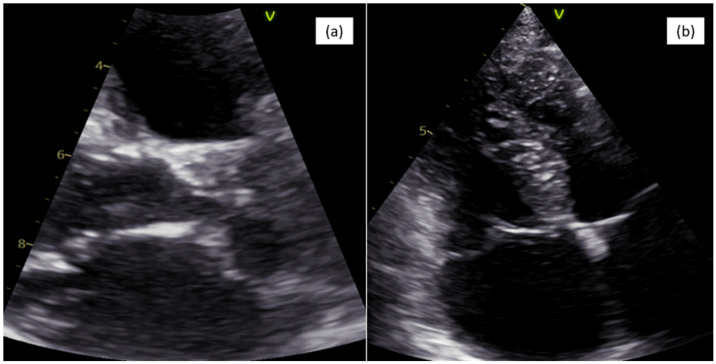
Echocardiography image: (**a**) parasternal long-axis view (PLAX) of unroofed coronary sinus and (**b**) right cavity enlargement.

The ECG showed a sinus rhythm, with a right axis deviation and right bundle block aspect. The chest X-ray described increased pulmonary circulation and mild cardiomegaly. Transthoracic 2D echocardiography revealed a large atrial septal defect with right atrial and ventricular overload with unroofed coronary sinus draining into the left atrium and persistent left superior vena cava, as presented in Figure 1. Considering the significantly dilated right cavities, a non-turbulent left–right shunt at the interatrial septum (IAS) and the inferior vena cava (IVC) with a diameter of approximately 2 cm is evident from the subcostal view with difficulty, suggestive of an ASD of the lower venous sinus. At the left atrial level, at the junction with the left auricle, an intracavity membrane is evident and appears to have continuity with the coronary sinus wall towards the left atrial. The upper wall of the coronary sinus is not visible. The coronary sinus is severely dilated with a diameter of 3.5 cm and without the upper wall. The injection of agitated saline into the left-side brachial vein highlights the severe dilation of the coronary sinus, persistent left superior vena cava and unroofed coronary sinus toward the left atrium. When injecting into the right sided brachial vein, a washing effect is evident, confirming the left–right shunt through the atrial septal defect. The right cavities are dilated with moderate tricuspid regurgitation, impairment of the systolic function of the right ventricle and a moderate degree of pulmonary hypertension (PAPS 57 mmHg). The aorta showed no malposition near the interventricular septum and without enlargement of the aortic root. There is a suture patch at the level of the peri-membranous interventricular septal defect, closing the ventricular septal defect with a length of 1.7 cm, without any residual shunt. This is associated with pulmonary mild stenosis and mild regurgitation.

**Figure 2:**
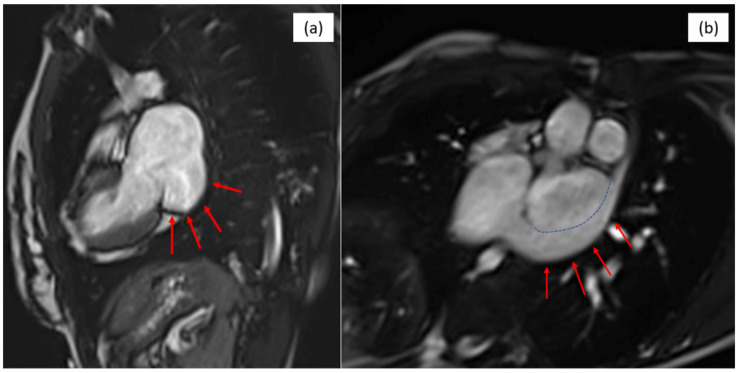
MRI images of (**a**) the unroofed coronary sinus (red arrows) and (**b**) left superior vena cava (red arrows) and demarcation between the coronary sinus and the left superior vena cava (blue dashed line).

In addition, cardiac magnetic resonance imaging (MRI) confirms a large atrial septal defect with an unroofed coronary sinus and persistent left superior vena cava, as shown in Figure 2. It describes an important left to right shunt with a ratio of pulmonary-to-systemic flow Qp/Qs of 2.8, a severely dilated right ventricle (tele-diastolic volume 152 mL/sq) with normal systolic function (RVEF of 62%) and a small aneurysm in the right ventricle (that may be a result of the infundibuloplasty).

The initial differential diagnosis included a residual ventricular septal defect, tricuspid regurgitation, pulmonary regurgitation or a left to right atrial shunt due to an atrial septal defect. Taking into consideration the enlargement of the right cavities with pulmonary hypertension in a symptomatic patient with an atrial septal defect–unroofed coronary sinus—type 1, the patient was referred to the cardiovascular surgical department for surgical treatment.

According to the ESC guideline, in patients with atrial septal defects and RV volume overload, the interventional or surgical closure of the defect is recommended regardless of symptoms (IB).

The patient was then adequately prepared for surgery and we performed an interatrial compartmentation with a bovine patch and tricuspid annuloplasty using an Edwards Lifescience 34 ring (Edwards Lifesciences, Irvine, CA, USA).

**Figure 3:**
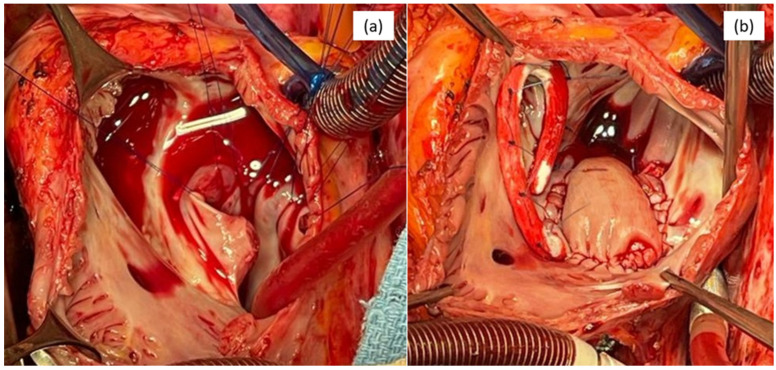
Intraoperative aspect: (**a**) right atrium and the defect—before repair and (**b**) patch and tricuspid ring—after repair.

The approach was through median sternotomy and right atriotomy. The surgery was performed via a cardio-pulmonary by-pass and St. Thomas antegrade cardioplegia. We used a 5-0 Prolene continuous suture for the patch, and Ti-Cron separate sutures were used for the tricuspid ring, with no peri-operatory incidents. The patient made a quick and safe recovery and was discharged on the fourth day, with no residual shunt. Figure 3 presents the intraoperative aspect of the right atrium and the defect before the repair and the final aspect after the repair.

## 3. Discussion

Raghib et al. first described this anomaly in 1965, reporting the association between an abnormal coronary sinus (CS) and a persistent left superior vena cava (PLSVC) draining directly into the left atrium, a constellation that later became known as Raghib syndrome [7]. This developmental defect results from a failure in the normal incorporation of the coronary sinus into the right atrium, leading to partial or complete unroofing and abnormal systemic venous return.

From a clinical standpoint, an unroofed CS with PLSVC may remain entirely asymptomatic for prolonged periods and is often discovered incidentally during imaging or surgery for other congenital heart defects [8]. However, when the left superior vena cava drains into the left atrium, a right-to-left shunt is created, which carries important pathophysiological and clinical implications. Such shunting may predispose patients to paradoxical systemic embolization, including ischemic stroke or a cerebral abscess, due to the direct passage of venous emboli into the systemic circulation. Less commonly, depending on the degree of shunting and associated defects, altered intracardiac flow may lead to chamber enlargement and, in rare cases, right ventricular volume overload and heart failure. Consequently, despite its potential clinical silence, an unroofed CS associated with PLSVC represents a diagnostically and prognostically significant anomaly that warrants careful recognition, particularly in patients with complex congenital heart disease [9,10].

Evidence from surgical series indicates that a type I unroofed coronary sinus—defined as complete unroofing associated with a persistent left superior vena cava—is the most prevalent subtype. In a cohort of 36 patients with unroofed coronary sinus syndrome, this form accounted for 41.7% of cases, followed by the partial unroofing of the terminal portion (type IV) in 30.6%, partial unroofing of the mid portion (type III) in 16.7%, and complete unroofing without a persistent left superior vena cava (type II) in 11.1% [3].

Although an unroofed coronary sinus is itself a rare atrial-level shunt, accounting for less than 1% of atrial septal defects and approximately 0.1% of congenital heart disease, its coexistence with Tetralogy of Fallot is exceptionally uncommon [7]. To date, this association has been documented almost exclusively through isolated case reports, including a single well-documented adult case and a very limited number of pediatric reports. While the subsequent literature has occasionally cited additional TOF-associated UCS or coronary sinus septal defect cases, these remain sporadic and anecdotal, with no dedicated case series or epidemiological data available, underscoring that the current evidence is confined to a case report level.

Table 1 summarizes the previously reported cases of Tetralogy of Fallot associated with an unroofed coronary sinus and related systemic venous anomalies. The available reports include both pediatric and adult patients, with ages ranging from early childhood to the fifth decade of life, although demographic data were incomplete in some cases.

In an adult, Jian et al. described a 41-year-old man with TOF and dextrocardia in whom a coronary sinus-type atrial septal defect and a partially unroofed coronary sinus coexisted with a persistent left superior vena cava (PLSVC). Surgical management was a single-stage total correction, consisting of the closure of the coronary sinus ASD using a pericardial patch with the redirection of the PLSVC into the right atrium, combined with a definitive TOF repair (ventricular septation with a Dacron patch and conotruncal repair) [5].

In contrast, the pediatric experience is even more limited and suggests that a UCS may appear as part of a broader atrial/venous developmental phenotype rather than an isolated pairing with TOF. Nomura et al. reported a rare pediatric surgical case in which TOF and a UCS occurred together with cor triatriatum and PLSVC. The repair required the resection of the intra-left atrial fibrous membrane (cor triatriatum), reconstruction of the LSVC return to the right atrium using a PTFE graft, atrial partition with a bovine pericardial patch, and standard conotruncal repair for TOF. Postoperative catheterization at 6 months demonstrated smooth LSVC drainage into the right atrium without a pressure gradient, and the clinical course was again uneventful [4]. A separate pediatric report by Malakan Rad et al. focused primarily on diagnosis rather than the surgical technique, describing a 4-year-old girl with TOF and a partially unroofed coronary sinus in the setting of bilateral SVC, diagnosed using contrast echocardiography with agitated saline and agitated 5% dextrose (DW5%). The authors emphasized DW5% as an effective and widely available ultrasound-enhancing approach without saline-related volume expansion [6].

Similarly to our case, Ueno et al. [11] reported the exceptionally rare coexistence of cor triatriatum, a partially unroofed coronary sinus, and a persistent left superior vena cava in a patient with a history of repaired Tetralogy of Fallot. Notably, a definitive surgical correction was performed 35 years after the initial operation for Tetralogy of Fallot. This delayed presentation underscores the importance of lifelong follow-up in patients with repaired complex congenital heart disease. The tailored surgical reconstruction included the resection of the left atrial membrane and the redirection of anomalous venous return. This case emphasizes the need for heightened awareness of atrial and systemic venous anomalies in patients with Tetralogy of Fallot, particularly in the setting of late-onset symptoms.

The diagnosis of a UCS can be a challenge. Transthoracic echocardiography is the first line of imaging used for suspected cases of coronary sinus congenital defects. Transthoracic echocardiography revealed the atrial septal defect of the lower venous sinus—suggestive of an unroofed coronary sinus. A CS is a posterior structure of the heart, so it is difficult to visualize from 2D echocardiography, with transesophageal echocardiography being a more useful tool to evaluate the CS [12]. A case series of 23 patients that comprised totally unroofed CSs (60.9%) and partially unroofed CSs in the mid-portion (26.2%) and in the terminal portion (17.4%) showed that 56.5% of the patients were correctly diagnosed by using transthoracic echocardiography [13].

Cardiac CT and cardiovascular magnetic resonance (CMR) are important tools to further evaluate the anatomy and functionality of the coronary sinus, providing a lot of information to interventional cardiologists and cardiovascular surgeons for the surgical approach. Moreover, CMR is noninvasive and does not use contrast agents or ionization radiation and is more recommended for children and young patients [14]. Hoppe et al. [15] studied 37 patients with atrial septal defects and revealed that 36 of them were detected using CMR and transthoracic echocardiography, while transthoracic echocardiography only detected 24 atrial septal defects from the 37 [14].

A normal coronary sinus caliber is 8.7 ± 2.5 mm. A previous surgical study with 24 patients showed that an unroofed CS is associated with other congenital anomalies in 100% of cases, about 75% with PLSVC [16]. A dilated CS was the first finding in our case that raised the suspicion of a PLSVC, which was confirmed by a bubble study, using a saline contrast agent injected into the left arm to enter directly in the left atrium.

The treatment of the unroofed coronary sinus and persistent superior vena cava consists of the closure of the defect, which is classically a surgical correction. This can be t can be either using a pericardial patch or direct sutures with the coronary sinus on the right. Direct suture of the defect is a feasible option when there is lack of tension to the surrounding structures, but the safest way is using pericardial patch, with the preferred one being the bovine patch [17,18,19].

The classical surgical approach is through a median sternotomy, already proven to be safe and effective. Other feasible options are the minimally invasive, endoscopic and robotic techniques but also a percutaneous repair when such an approach is possible. The minimally invasive technique requires the use of endoscopic visualization, as the lesion is only visible endoscopically and guided by previous 3D transesophageal echocardiography [20]. The current trend is towards reduced invasiveness and leads to an improved outcome for the patient [21].

The robotic endoscopic method was used in a small number of cases, and even though the cosmetic results and postoperative recovery time were improved, further studies are needed to evaluate the safety and cost–benefit balance of such a method, as it is known to be expensive and has a slow learning curve [22].

Percutaneous repair is a recent approach to the phenomenon discussed, with different complications. Occluding devices implanted in this fashion have limitations regarding the diameter of the defect and the proximity to the surrounding structures. Further studies need to develop the safety and applicability of this approach [23].

Systemic venous anomalies such as an unroofed coronary sinus may be easily overlooked at the time of the initial surgical repair for Tetralogy of Fallot, particularly during early childhood. Historically, diagnostic imaging techniques available during infancy—especially in earlier surgical eras—had a limited spatial resolution and a reduced ability to adequately visualize posterior cardiac structures, including the coronary sinus and systemic venous drainage [24,25]. Furthermore, during complex neonatal or infant cardiac surgeries, clinical and surgical priorities are primarily directed toward correcting major intracardiac lesions, such as ventricular septal defects and right ventricular outflow tract obstruction, which may lead to associated venous anomalies being considered hemodynamically insignificant at that stage and therefore remaining undetected [26]. Consequently, an unroofed coronary sinus may remain clinically silent for years or decades and only become apparent later in life, once the shunt magnitude increases or secondary hemodynamic consequences develop [24].

## 4. Conclusions

This case highlights the exceptional rarity and diagnostic complexity of the association between an unroofed coronary sinus (UCS) and Tetralogy of Fallot (TOF), particularly in adults with previously repaired congenital heart disease. Our report illustrates that a UCS associated with a persistent left superior vena cava (PLSVC) may remain occult for decades, even after a complex surgical correction of TOF in infancy, and may only become clinically apparent later in adulthood when hemodynamic consequences evolve. This case underscores the importance of a comprehensive reassessment of the systemic venous and atrial anatomy in symptomatic adults with repaired TOF, using advanced multimodality imaging. Given that the existing evidence is limited to isolated case reports, this observation contributes novel data to the literature and reinforces the need for a heightened awareness of rare venous anomalies during the long-term follow-up of congenital heart disease patients.

## Figures and Tables

**Table 1:** Published cases of Tetralogy of Fallot associated with unroofed coronary sinus.

Author	Age/Sex	Diagnostic	Treatment	Outcome
Jian Z et al. [5], 2010	41 y/male	2DTTE/CT scan	Closure of the coronary sinus atrial septal defect with PLSVC entering the right atrium using a pericardial graft and ventricular partition with a Dacron patch and conotruncal repair for Tetralogy of Fallot.	Uneventful
Nomura K et al. [3], 1997	NS	CT scan	Resection of the intra-left atrial fibrous membrane, reconstruction of the LSVC return pathway to the right atrium with PTFE graft, atrial partition with a bovine pericardial patch and the conotruncal repair for Tetralogy of Fallot.	Uneventful
Malakan Rad E et al. [6], 2019	4 y/female	Dextrose 5% TTE	NS.	NS
Ueno T et al. [11], 2008	40 y/female	TTE	Resection of the left atrial membrane and redirection of anomalous venous return.	Uneventful

TTE: transthoracic echocardiography; NS: not specified; and CT: computed tomography.

## Data Availability

The raw data supporting the conclusions of this article will be made available by the authors on request.
